# Micrococcal nuclease regulates biofilm formation and dispersal in methicillin-resistant *Staphylococcus aureus* USA300

**DOI:** 10.1128/msphere.00126-24

**Published:** 2024-05-02

**Authors:** Jeffrey B. Kaplan, Alexander R. Horswill

**Affiliations:** 1 Department of Biology, American University, Washington, DC, USA; 2 Department of Immunology and Microbiology, University of Colorado Anschutz Medical Campus, Aurora, Colorado, USA; University of Kentucky College of Medicine, Lexington, Kentucky, USA

**Keywords:** Nuc1, Nuc2, sub-MIC, thermonuclease, clumping dispersal

## Abstract

Biofilm formation is an important virulence factor for methicillin-resistant *Staphylococcus aureus* (MRSA). The extracellular matrix of MRSA biofilms contains significant amounts of double-stranded DNA that hold the biofilm together. MRSA cells secrete micrococcal nuclease (Nuc1), which degrades double-stranded DNA. In this study, we used standard methodologies to investigate the role of Nuc1 in MRSA biofilm formation and dispersal. We quantified biofilm formation and extracellular DNA (eDNA) levels in broth and agar cultures. In some experiments, cultures were supplemented with sub-MIC amoxicillin to induce biofilm formation. Biofilm erosion was quantitated by culturing biofilms on rods and enumerating detached colony-forming units (CFUs), and biofilm sloughing was investigated by perfusing biofilms cultured in glass tubes with fresh broth and measuring the sizes of the detached cell aggregates. We found that an MRSA *nuc1^−^* mutant strain produced significantly more biofilm and more eDNA than a wild-type strain, both in the absence and presence of sub-MIC amoxicillin. *nuc1^−^* mutant biofilms grown on rods detached significantly less than wild-type biofilms. Detachment was restored by exogenous DNase or complementing the *nuc1^−^* mutant. In the sloughing assay, *nuc1^−^* mutant biofilms released cell aggregates that were significantly larger than those released by wild-type biofilms. Our results suggest that Nuc1 modulates biofilm formation, biofilm detachment, and the sizes of detached cell aggregates. These processes may play a role in the spread and subsequent survival of MRSA biofilms during biofilm-related infections.

**IMPORTANCE:**

Infections caused by antibiotic-resistant bacteria known as methicillin-resistant *Staphylococcus aureus* (MRSA) are a significant problem in hospitals. MRSA forms adherent biofilms on implanted medical devices such as catheters and breathing tubes. Bacteria can detach from biofilms on these devices and spread to other parts of the body such as the blood or lungs, where they can cause life-threatening infections. In this article, researchers show that MRSA secretes an enzyme known as thermonuclease that causes bacteria to detach from the biofilm. This is important because understanding the mechanism by which MRSA detaches from biofilms could lead to the development of procedures to mitigate the problem.

## INTRODUCTION

*Staphylococcus aureus* is a major public health burden in both community and hospital settings (1). In hospitals, *S. aureus* is a major cause of infections associated with implanted medical devices because of the ability of most *S. aureus* strains to form biofilms on biomaterial surfaces (2). *S. aureus* biofilms on implanted devices can act as a nidus of infection when bacterial cells detach from the biofilm and spread to other parts of the body such as the blood, lung, or bladder (3). In addition, nearly half of all *S. aureus* hospital isolates are methicillin-resistant *S. aureus* (MRSA), a multidrug-resistant strain that further complicates treatment of these common nosocomial infections (4).

*S. aureus* secretes numerous virulence factors into its surrounding environment, which contribute to host colonization and virulence. These include extracellular enzymes, pore-forming toxins, and superantigens (5). Among these is micrococcal nuclease (Nuc1), also known as thermonuclease, a secreted, Ca^2+^-dependent, thermostable phosphodiesterase that hydrolyzes both RNA and DNA (6). Nuc1 is produced by all strains of *S. aureus. nuc1^−^* mutant strains exhibit decreased survival in mouse models of peritonitis (6) and intranasal infection (7). Nuc1 may contribute to virulence *in vivo* by facilitating escape from neutrophil extracellular traps (7–9). Nuc1, along with other exoenzymes such as proteases, lipases, and hyaluronidases, may also contribute to tissue invasion or nutrient acquisition *in vivo* (10). All strains of *S. aureus* also produce a second extracellular nuclease termed Nuc2 (11, 12). Like Nuc1, the homologous Nuc2 enzyme is Ca^2+^-dependent, thermostable, and is able to hydrolyze both DNA and RNA (11, 12). Nuc2 is expressed *in vivo*, and purified Nuc2 protein inhibits *S. aureus* biofilm formation in microtiter plate wells and detaches preformed biofilms (11), suggesting that it may also play a role in biofilm formation and dispersal. However, *nuc1* and *nuc2* genes are located in different regions of the *S. aureus* chromosome, and the Nuc2 protein remains bound to the cell surface (11, 12).

Proteins and double-stranded DNA are the most common extracellular components of biofilms produced by most MRSA strains (13). Extracellular DNA (eDNA) consists of genomic DNA released by lysis of a subpopulation of cells within the biofilm (3, 14). eDNA may help stabilize the biofilm matrix by binding to secreted eDNA-binding proteins and membrane-attached lipoproteins (15) or to extracellular poly-*N*-acetylglucosamine polysaccharides (16). Previous studies have shown that biofilms produced by *nuc1^−^* mutant strains contain more eDNA than wild-type (WT) biofilms (17) and that biofilm formation is enhanced in *nuc1^−^* mutant strains (14, 17). In addition, expression of *nuc1* is repressed during biofilm formation (6, 17). These findings suggest that exogenous Nuc1 can degrade eDNA, thereby decreasing the adhesive properties of the biofilm matrix.

In the present study, we investigated the role of Nuc1 and Nuc2 in biofilm formation using the MRSA strain LAC, a USA300 clone. By comparing a WT strain to *nuc1^−^*, *nuc2^−^*, and *nuc1^−^*/*nuc2^−^* mutant strains, we found that Nuc1, but not Nuc2, modulates the amount of eDNA in the biofilm matrix, the adhesiveness of the biofilms, and the amount of the biofilm formed, which is induced by low-dose amoxicillin. We further investigated the role of Nuc1 in two different mechanisms of biofilm dispersal, namely, erosion and sloughing. Biofilm erosion refers to the release of single cells or small clusters of cells from a biofilm, whereas sloughing refers to the detachment of large portions of the biofilm (18). Results from assays comparing biofilm dispersal in WT and *nuc1^−^* mutant strains suggests that Nuc1 not only mediates detachment of cells from MRSA biofilms but also modulates the sizes of the detached cell aggregates.

## MATERIALS AND METHODS

### Bacterial strains, media, and growth conditions

The bacterial strains used in this study are listed in Table 1. Bacteria were cultured on Tryptic Soy Agar plates or in filter-sterilized tryptic soy broth (BD Diagnostics). All cultures were incubated at 37°C. For plasmid-harboring strains, media were supplemented with 10 µg/mL chloramphenicol. To induce biofilm formation, media were supplemented with sub-MIC concentrations of amoxicillin ranging from 0.002 to 2 µg/mL. To induce biofilm detachment, the broth was supplemented with 10 µg/mL recombinant human DNase I (Genentech), which was previously shown to efficiently detach *S. aureus* biofilms from polystyrene microtiter plate wells (19). Bacterial inocula were prepared in sterile broth from 24-h-old agar colonies and then passed through a 5-µm-pore-size syringe filter to enrich for single cells, as previously described (20).

**TABLE 1:** *S. aureus* strains used in this work

Strain	Relevant characteristics	Reference
AH1263	WT CA-MRSA USA300 strain LAC (Erm^S^ variant)	(21)
AH1680	AH1263 *nuc1* (Nuc1*^−^* mutant)	(17)
AH3057	AH1263 *nuc2* (Nuc2*^−^* mutant)	(11)
AH3051	AH1263 *nuc1 nuc2* (Nuc1*^−^*/Nuc2*^−^* double mutant)	(11)
AH1787	AH1680 carrying plasmid pCM28 (empty vector control)	(7)
AH1773	AH1680 carrying plasmid pCM28*nuc1* (Nuc1 complementing vector)	(7)

### Six-well microtiter plate biofilm assay

Aliquots of inocula (4 mL each; ca. 10^2^ CFU/mL) were transferred to the wells of a 6-well microtiter plate (Falcon #353046), and the plate was incubated statically under conditions of low vibration, as previously described (22). After 18 h, a 1 cm^2^ area in the center of each well was photographed. Wells were then rinsed with water and stained for 1 min with 4 mL of Gram’s crystal violet. Wells were rinsed with water and dried, and the same area of each well was rephotographed.

### Ninety-six-well microtiter plate biofilm assay

Aliquots of inocula (180 µL each; 10^5^–10^6^ CFU/mL) were transferred to the wells of a 96-well microtiter plate (Corning #3599) containing 20 µL of an antibiotic dissolved in water at a concentration equal to 10 times the desired final concentration. Control wells were filled with 180 µL of the inoculum and 20 µL of water or 180 µL of sterile broth and 20 µL of water. The plates were incubated for 18 h. The amount of biofilm biomass in each well was quantitated by rinsing the wells with water and staining for 1 min with 200 µL of Gram’s crystal violet. The wells were then rinsed with water and dried. The bound crystal violet was dissolved in 200 µL of 33% acetic acid and quantitated by measuring the absorbance of the wells at 620 nm.

### Isolation and analysis of extracellular DNA

Extracellular DNA was isolated from lawns of bacteria cultured on agar, also known as colony biofilms, using the method described by Karwacki et al*.* (23). Briefly, 100-µL aliquots of the inoculum (>10^8^ CFU) was spread onto agar plates supplemented with 0 or 0.2 µg/mL amoxicillin. After incubation for 24 h, the cell paste from each plate was transferred to a separate, preweighed 1.5-mL microcentrifuge tube, weighed, and resuspended in TE buffer at a concentration of 1 mg/mL. The sample was mixed by vortex agitation for 10 min, and the cells were pelleted by centrifugation. The supernatant was sterilized by passage through a 0.2-μm-pore-size filter. A 20 µL volume of the colony biofilm extract was analyzed by agarose gel electrophoresis, and the DNA was visualized by staining with ethidium bromide.

### Biofilm erosion assay

**Fig 1:**
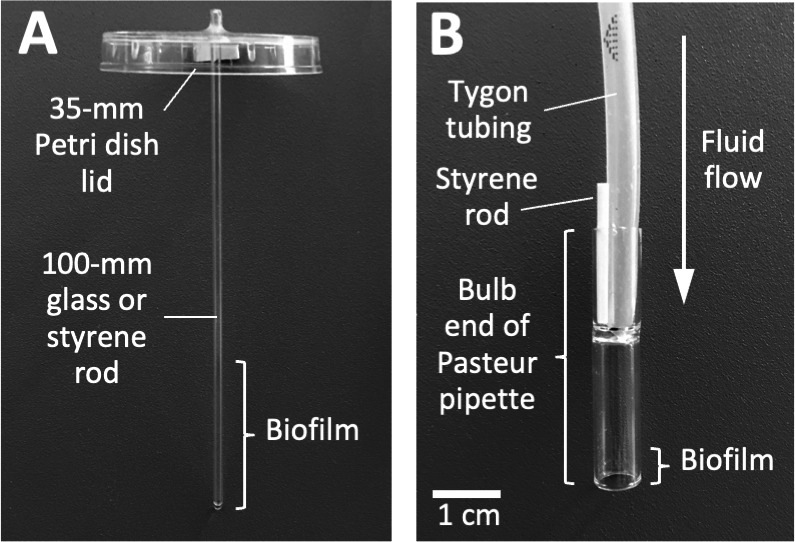
Apparatus used to measure biofilm erosion (**A**) and biofilm sloughing (**B**). The construction and use of these apparatus are presented in Materials and Methods.

An apparatus to measure biofilm erosion under static conditions (Fig. 1A) was constructed and employed as follows. First, a 2-mm-diameter hole was drilled in the center of a 35-mm-diam Petri dish lid (Sarstedt # 83.1800.001). Next, a 100 mm polystyrene or glass rod was placed in the hole and secured in a vertical position with hot-melt adhesive. The polystyrene rods (1.5 mm diameter) were purchased from Plastruct (City of Industry CA), and the glass rods were made from 1.9-mm-diameter open-one-end capillary tubes (Stuart Glass # EW-03013-64) with the closed end facing down. The rods were positioned so that 5 mm protruded from the top of the lid (Fig. 1A). The apparatus were sterilized with 70% ethanol, air-dried, and then placed on top of 50-mL conical centrifuge tubes (Corning # 430290) containing 20 mL of the bacterial inoculum at 10^5^–10^6^ CFU/mL. Approximately 30 mm of the rod or capillary tube was suspended in the broth. After incubation for 16 h, the apparatus were carefully transferred to fresh tubes containing 25 mL of prewarmed sterile broth and incubated for 1 min to detach loosely adherent cells. The apparatus were then transferred to fresh tubes containing 25 mL of prewarmed sterile broth. After 2 h, the apparatus were removed from the centrifuge tubes. To enumerate the CFUs in the broth, the broth was mixed by vortex agitation for 30 s, serially diluted in phosphate-buffered saline (PBS), and then plated on agar for CFU enumeration. To enumerate the CFUs on the rods, the rods were detached from the Petri dish lids, placed in 15-mL conical centrifuge tubes containing 10 mL of PBS, and sonicated for 30 s at 40% duty cycle in an ultrasonic homogenizer equipped with a 1/8 in. probe (Fisher Scientific model 505). Sonicates were serially diluted in PBS and plated on agar for CFU enumeration. The fraction of detached cells (percent CFUs in broth) was calculated by dividing the total number of CFUs in the broth by the total number of CFUs in the broth plus the total number of CFUs on the rod. Assays were performed in duplicate to quadruplicate tubes for each experimental condition.

### Biofilm sloughing assay

An apparatus to measure biofilm sloughing under flow conditions (Fig. 1B) was constructed and employed as follows. First, biofilm reactors were constructed by cutting 38-mm-long sections from the bulb ends of standard 9-in. flint glass Pasteur pipettes (VWR # 14672-380) by using a glass cutter. The glass reactors were rinsed in 1% HCl for 2 h, rinsed with 70% ethanol and ultrapure water, and then air-dried. Glass reactors were inoculated by standing them (bulb-side-down) in a 6-well microtiter plate well containing 2 mL of the bacterial inoculum at 10^5^–10^6^ CFU/mL, which brought the bottom 5 mm of each reactor in contact with the inoculum. After 10 min, the reactors were connected to 1/8 × 3/16 in. silicon tubing (Tygon 3350 #ABW00006) and perfused with fresh broth for 48 h at a rate of 5–7 mL/h. A short 1.5-mm-diameter polystyrene rod (Fig. 1B) functioned to secure the tubing to the glass reactor and to form a vent that allowed the reactor to act as a continuous drip chamber. A total of four reactors were perfused in each experiment, two inoculated with the WT strain and two with the *nuc1^−^* mutant strain. To enumerate CFUs in the eluate, drops were collected directly into 1.5-mL microcentrifuge tubes, mixed by vortex agitation for 30 s, serially diluted in PBS, and plated on agar for CFU enumeration. To measure the sizes of the detached cell aggregates, drops were collected directly onto glass microscope slides and then fixed, stained with Gram’s crystal violet, and photographed at 400× under an inverted microscope. For each strain, the distribution of particle sizes in five random microscopic fields was analyzed using ImageJ software. For direct visualization of detached cell aggregates, drops of the eluate were collected into the wells of a 6-well microtiter plate and then photographed on a dark background.

### Statistics and reproducibility of results

Biofilm erosion assays were performed in triplicate tubes or quadruplicate tubes and were repeated two to four times. The significance of differences between percent CFUs in broth values was calculated using a two-tailed Student’s *t*-test for pairwise comparisons and a one-way ANOVA with Tukey’s post hoc analysis for comparison of more than two groups. A *P* value of <0.05 was considered significant. Biofilm sloughing assays were performed in duplicate reactors for each strain (wild-type and *nuc1^−^*) and were repeated twice. The significance of differences between particle size distributions in the sloughing assay was calculated using a two-way chi squared test.

## RESULTS

### Biofilm formation by WT and nuclease mutant strains in 6-well microtiter plate wells

**Fig 2:**
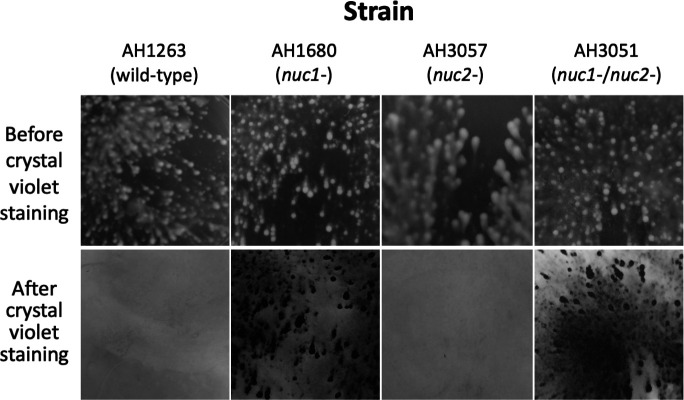
Biofilm formation by MRSA USA300 WT and nuclease mutant strains in 6-well tissue culture-treated microplates under conditions of low vibration. Top panels show surface-attached growth after incubation for 16 h. Bottom panels show the identical areas of the plate after they were rinsed with water and stained with crystal violet. The size of each imaged area is 1 cm^2^. The 6-well microplate assay was performed three times using four to six wells for each strain in each experiment. All four strains exhibited the same biofilm growth and crystal violet detachment phenotypes in all wells in each experiment. Representative wells are shown above.

MRSA WT, *nuc1^−^*, *nuc2^−^*, and *nuc1^−^*/*nuc2^−^* strains were cultured in broth in 6-well microtiter plates at low densities and under conditions of low vibration. After 18 h, the wells were photographed (Fig. 2, top panels). All four strains produced surface-attached biofilms under these conditions. *nuc1^−^* and *nuc1^−^*/*nuc2^−^* biofilms appeared denser and more compact than those of the WT and *nuc2^−^* strains. After rinsing the wells with water and staining with crystal violet, biofilms of the *nuc1^−^* and *nuc1^−^*/*nuc2^−^* strains remained firmly attached to the surface, whereas biofilms of the WT and *nuc2^−^* strains were readily detached (Fig. 2, bottom panels). These results suggest that Nuc1, but not Nuc2, contributes to MRSA USA300 biofilm formation under these conditions.

### Induction of biofilm formation by sub-MIC amoxicillin in WT and nuclease mutant strains

**Fig 3:**
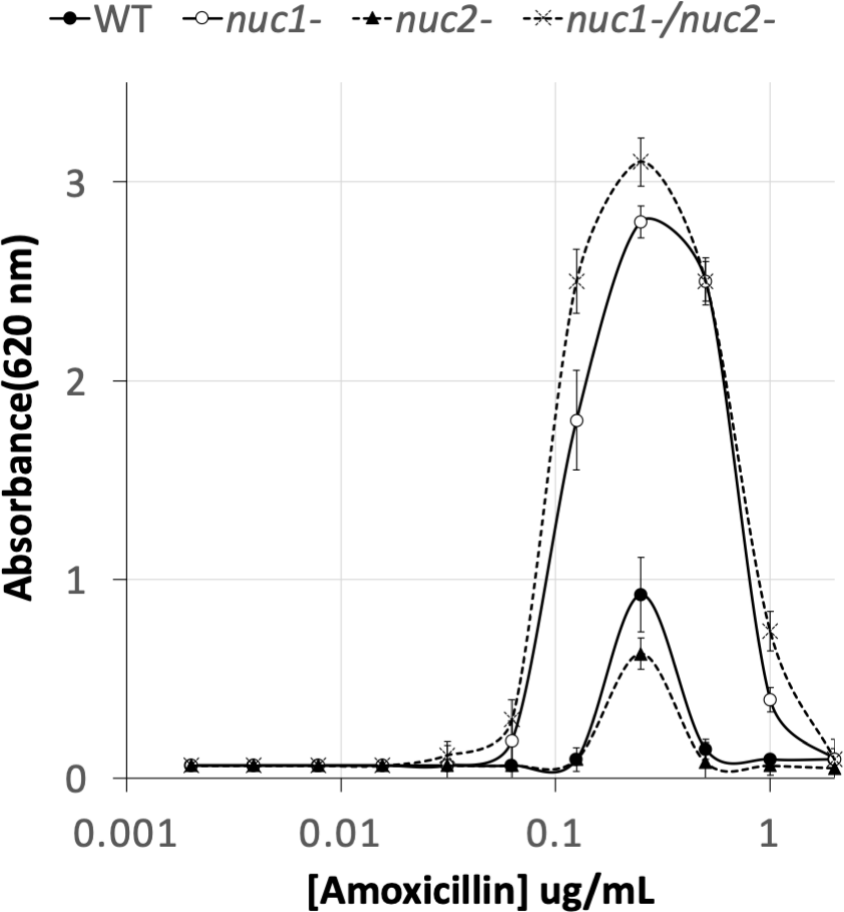
Biofilm formation by MRSA USA300 WT and nuclease mutant strains in 96-well microtiter plates in the presence of increasing concentrations of sub-MIC amoxicillin. Absorbance at 620 nm is proportional to biofilm biomass. Values show mean and range for duplicate wells. The biofilm induction assay was performed three times using duplicate wells for each strain. Duplicate wells exhibited an average variation of 7% within each experiment and an average of 18% between experiments. In all three experiments, the dose–response curves (antibiotic concentration, amplitude of stimulus response, and range of the stimulatory dose) were the same for all four strains tested. Representative graphs from one experiment are shown above.

Previous studies showed that low doses of amoxicillin induce biofilm formation in MRSA USA300 by a mechanism that involved autolysis and eDNA release (24, 25). To determine whether Nuc1 or Nuc2 plays a role in this process, we used a 96-well crystal violet binding assay to quantitate biofilm formation by WT, *nuc1^−^*, *nuc2^−^*, and *nuc1^−^*/*nuc2^−^* strains cultured in sub-MIC amoxicillin concentrations ranging from 0.002 to 2 µg/mL (Fig. 3). The MIC of amoxicillin against strain USA300 is >8 µg/mL (24). Low-dose amoxicillin induced biofilm formation in all four strains, with maximum induction occurring at a concentration of 0.2 µg/mL. However, biofilm induction occurred over a greater range of amoxicillin concentrations and resulted in a significantly greater amount of biofilm biomass in the *nuc1^−^* and *nuc1^−^*/*nuc2^−^* strains compared to the WT and *nuc2^−^* strains (Fig. 3). These results suggest that Nuc1, but not Nuc2, can modulate the structure of MRSA biofilms under both antibiotic-induced and uninduced conditions. These results are consistent with those of previous studies showing that nuclease activity is not required for β-lactam-induced MRSA biofilm formation (24).

### eDNA production in WT and nuclease mutant strains

**Fig 4:**
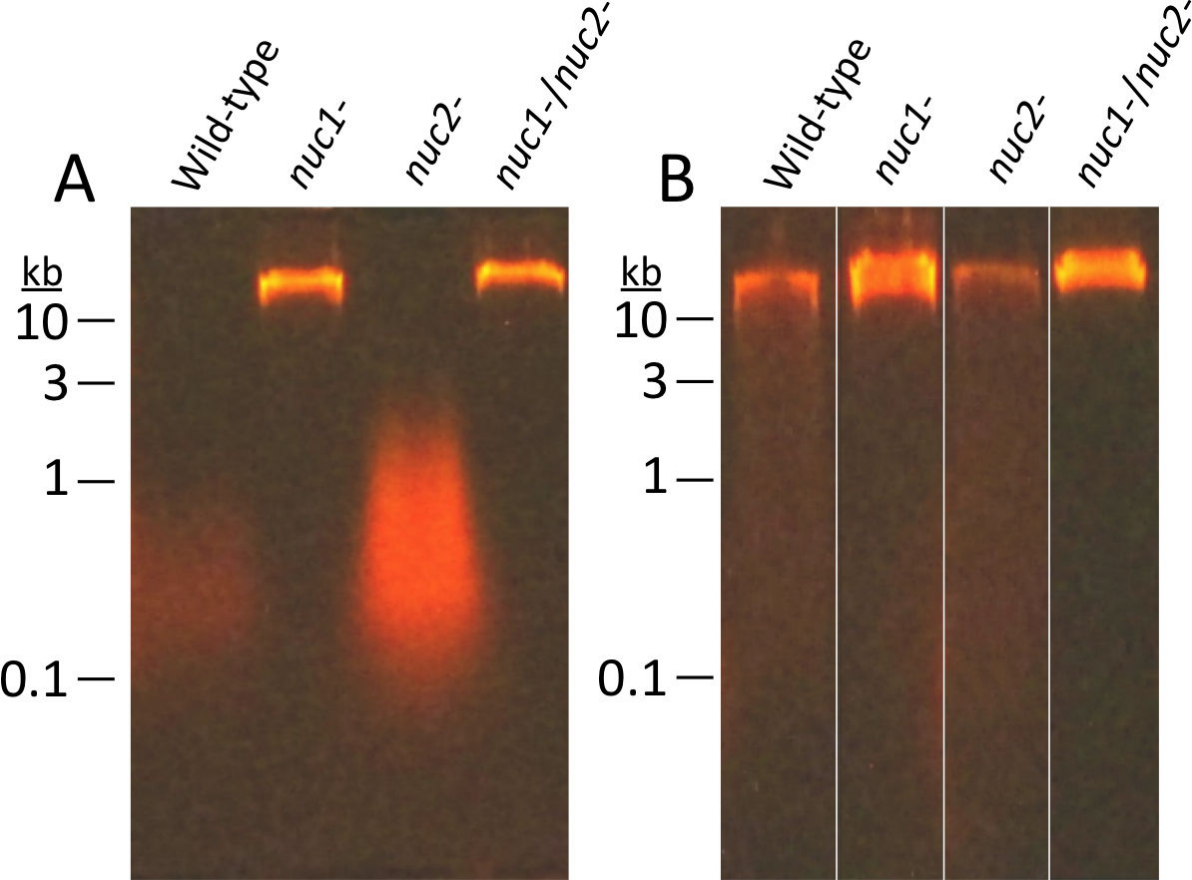
Agarose gel electrophoretic analysis of extracellular DNA (eDNA) produced by MRSA USA300 WT and nuclease mutant strains. eDNA was harvested from colony biofilms cultured in TSA (**A**) or TSA supplemented with 0.2 µg/mL amoxicillin (**B**). Numbers at the left indicate the sizes of molecular weight markers electrophoresed in an adjacent lane. eDNA was isolated and analyzed from three independent cultures of each strain for each condition (±antibiotic). In all cases, the amount and size of eDNA and its relative abundance among the strains exhibited the same pattern as those in the representative gel images shown above.

To investigate the role of Nuc1 and Nuc2 in MRSA USA300 eDNA production, we isolated eDNA from colony biofilms of WT, *nuc1^−^*, *nuc2^−^*, and *nuc1^−^*/*nuc2^−^* strains and isolated the DNA by agarose gel electrophoresis (Fig. 4A). The *nuc1^−^* and *nuc1^−^*/*nuc2^−^* strains produced significantly more high-molecular weight eDNA (> 25 kb) than the WT and *nuc2^−^* strains. All four strains produced high-molecular weight DNA in the presence of low-dose amoxicillin (Fig. 4B), which suggests that Nuc2 may regulate eDNA production under antibiotic-induced conditions. Taken together, our results demonstrate that high-molecular weight eDNA production inversely correlates with Nuc1 production (Fig. 4), but positively correlates with biofilm tenacity (Fig. 2) and antibiotic-induced biofilm formation (Fig. 3).

### Role of Nuc1 in biofilm erosion

We next investigated the role of Nuc1 in MRSA biofilm erosion. We focused on Nuc1 because it is the primary modulator of MRSA eDNA production and biofilm formation under uninduced conditions. Biofilm erosion was quantitated by culturing WT and *nuc1^−^* biofilms on polystyrene or glass rods. After 2 h, the rods were removed from the tubes and the number of CFUs on the rod and in the broth was measured. Percent biofilm dispersal was calculated as (broth CFUs)/(broth CFUs + rod CFUs) ×100. The total CFUs per tube ranged from 10^8^ to 10^9^ for both polystyrene and glass rods.

**Fig 5:**
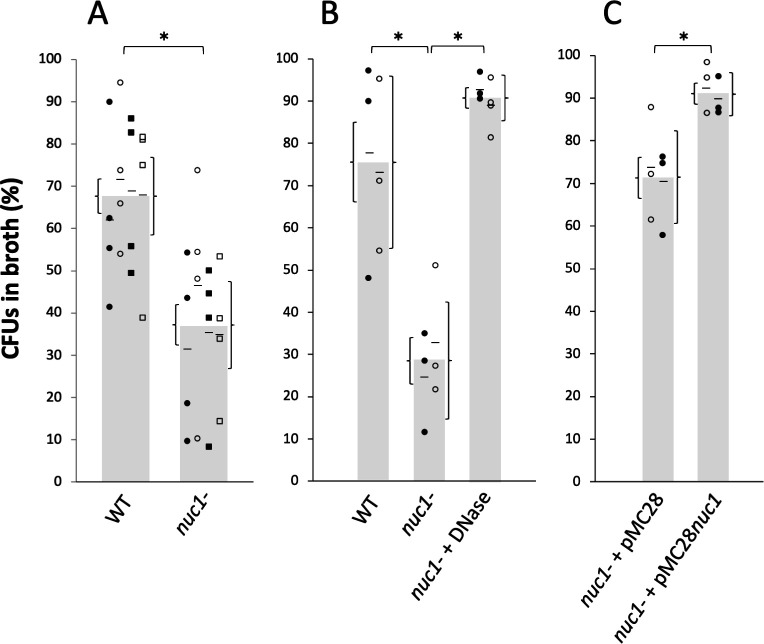
Erosion of MRSA USA300 wild-type (WT) and *nuc1^−^* mutant biofilms from polystyrene rods (A) and glass rods (B and C). Bars show the average percent of total CFUs in the broth after 2 h. Each symbol represents one rod. Results from individual experiments (four in panel A and two in panels B and C) are indicated by different symbols (filled and open circles and squares). Short horizontal bars indicate mean values for individual experiments. For panel A, there was a significant difference between the means of the four independent experiments, as determined by *t*-test (*P* = 0.0002), and no significant difference between the means of any pair of individual experiments for either the WT or *nuc1^−^* strains, as determined by one-way ANOVA with Tukey’s post hoc analysis (*P* = 0.889 for the WT strain and *P* = 0.743 for the *nuc1^−^* strain). Curly brackets to the left and right of the value bars indicate SE and 95% CI in panel A, respectively, and SE and SD in panels B and C, respectively. Asterisks denote groups with significantly different distributions (*P* < 0.001) based on *t*-tests for panels A and C and an ANOVA test for panel B. (**A**) Erosion of WT and *nuc1^−^* biofilms from polystyrene rods. (**B**) Erosion of WT and *nuc1^−^* biofilms from glass rods. Some *nuc1^−^* biofilms were incubated in broth supplemented with DNase. (**C**) Erosion of *nuc1^−^* biofilms harboring pMB28 (empty plasmid control) or pMB28*nuc1* (*nuc1* complementing plasmid) from glass rods.

Figure 5A shows the results from multiple biofilm erosion assays performed on polystyrene rods. On average, significantly more CFUs detached from WT biofilms compared to the number of CFUs that detached from *nuc1^−^* mutant biofilms (68% of CFUs in the broth for WT versus 38% for *nuc1^−^* mutant). The same erosion phenotype was observed when biofilms were cultured on glass rods (76% CFUs in broth for the WT versus 38% for *nuc1^−^* strains) (Fig. 5B). There was no significant difference between erosion rates from polystyrene or glass rods for either the WT strain (*P* = 0.37) or the *nuc1^−^* mutant strain (*P* = 0.37). However, significantly more number of CFUs were present in the broth when *nuc1^−^* mutant biofilms were allowed to erode from glass rods in broth supplemented with DNase compared to the no DNase control (Fig. 5B) or when the *nuc1^−^* strain carried a WT *nuc1* gene on the plasmid. (Fig. 5C). These findings confirm that Nuc1 mediates MRSA biofilm erosion.

### Role of Nuc1 in biofilm sloughing

**Fig 6:**
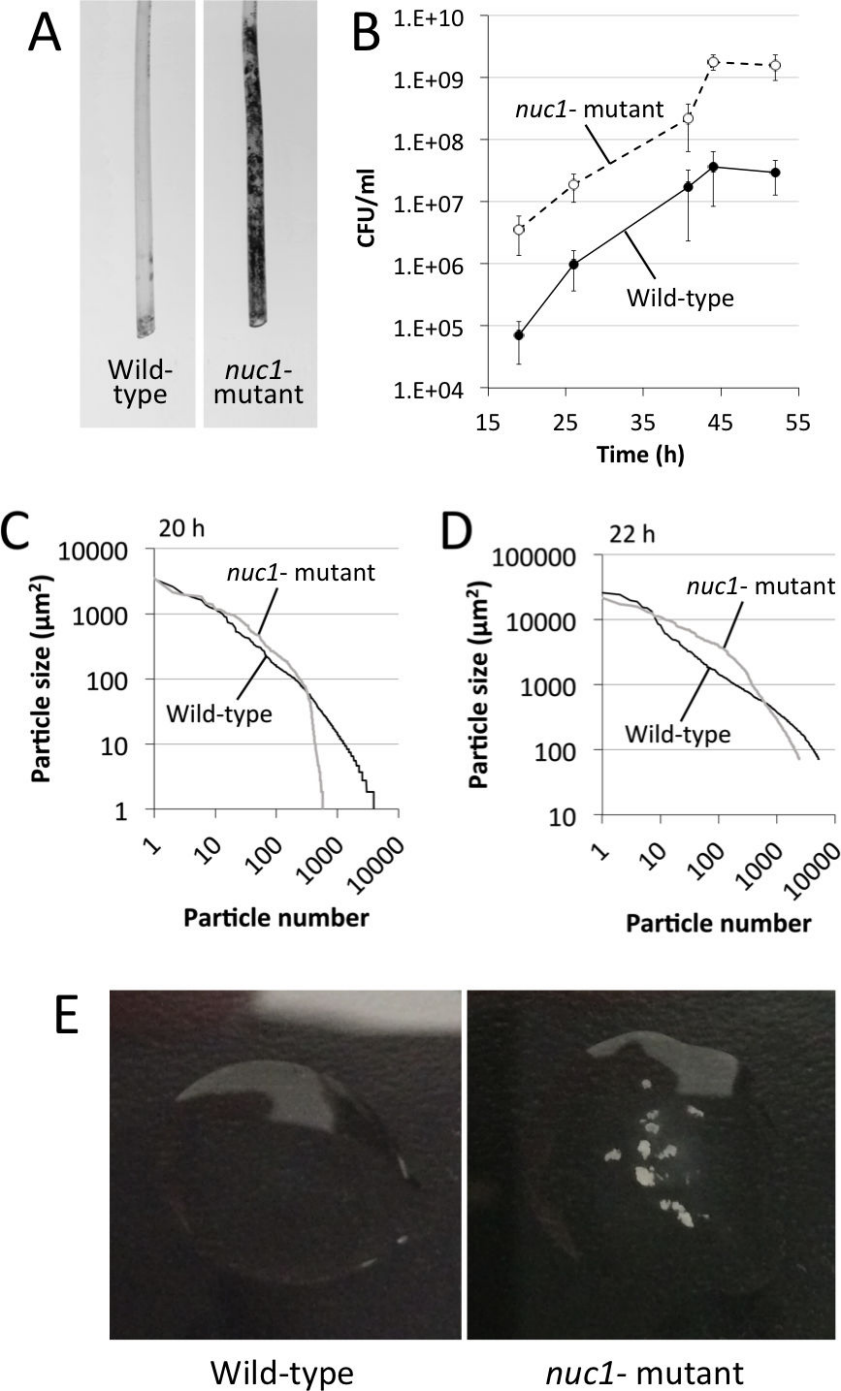
Biofilm sloughing assay. (**A**) Photographs of the sections of silicone tubing located directly above the glass biofilm reactors. The tubing sections were stained with crystal violet after 48 h of perfusion. (**B**) CFU/mL in the flowthrough from 17 to 52 h. Values show the mean and range for duplicate reactors. (**C and D**) Distribution of particle sizes in the eluates from two independent sloughing experiments. Eluates were analyzed after 20 h (C) or 22 h (D) of perfusion. (**E**) Photographs of drops of the eluate after 48 h of perfusion.

Biofilm sloughing is the detachment of large portions of a biofilm, which usually occurs in the later stages of biofilm formation (18). To quantitate biofilm sloughing, we grew WT and *nuc1^−^* mutant biofilms in glass tubes perfused with the fresh broth and measured the number of CFUs and sizes of detached cell aggregates in the eluate (Fig. 1B). Both strains formed biofilms in the glass tube reactors and dispersed. Over the course of the 48-h assay, the *nuc1^−^* mutant biofilm grew inside the silicone tubing that supplied fresh broth to the reactor as evidenced by crystal violet staining (Fig. 6A). Biofilms of both strains dispersed at high levels from 18 to 52 h (Fig. 6B), but *nuc1^−^* mutant biofilms released at least ten times more CFUs than WT biofilms at all time points tested, probably because of a larger amount of biofilm biomass.

We also measured the sizes of particles that detached from WT and *nuc1^−^* mutant biofilms using ImageJ software. Graphs showing the distribution of particle sizes from two independent sloughing experiments are shown in Fig. 6C and D. Characteristics of the detached particles from both experiments are summarized in Table 2. In both experiments, the distributions of particle sizes were significantly different (*P* < 0.0001; two-way chi squared test) (Fig. 6C and D). In addition, the average number of particles in the *nuc1^−^* eluate was significantly less than the number in the WT eluate, and the average particle size in the *nuc1^−^* eluate was significantly greater than that in the WT eluate in both experiments (Table 2). Differences between the number of particles, the average particle size, and the range of particle sizes between the two experiments shown in Fig. 6C and D and Table 2 may be due to the 2-h time difference or due to variations in the way in which the cells were fixed and stained or the manner in which the images were captured and analyzed on different days. After 48–52 h of perfusion, macroscopic clusters of cells were present in the *nuc1^−^* eluate, but not in the WT eluate (Fig. 6E).

**TABLE 2:** Characteristics of cell aggregates in the eluate of WT and *nuc1^−^* biofilms perfused with the broth for 20–22 h^*a*^

	Expt 1 (20 h)		Expt 2 (22 h)	
	AH1263 (WT)	AH1680 (*nuc1^−^*)	AH1263 (WT)	AH1680 (*nuc1^−^*)
Average number of particles (± SD) per microscopic field	1,436 ± 438	135 ± 33^*b*^	1,072 ± 287	492 ± 108^*b*^
Average particle size (µm^2^)	15	144^*b*^	317	723^*b*^

^*a*^ Results from two independent experiments are shown.

^*b*^ Significantly different from strain AH1263 (*P* < 0.01, two-tailed Student’s *t*-test).

## DISCUSSION

Previous studies showed that biofilms produced by MRSA *nuc1^−^* mutant strains are thicker and contain more eDNA than WT biofilms (14, 17, 25) and that *nuc1* expression is repressed during biofilm formation (6, 17). These findings are consistent with the hypothesis that Nuc1 degrades eDNA and decreases the adhesive properties of the biofilm matrix. A corollary hypothesis is that Nuc1 may function as an endogenous mediator of biofilm dispersal in this species. Dispersal of biofilms from indwelling medical devices is a clinically relevant process that may lead to invasive infections such as ventilator-associated pneumonia and endocarditis (26). Although Moormeier et al*.* (27) showed that Nuc1 mediates the detachment and dispersal of cells prior to the development of characteristic tower-like structures in the early stages of *S. aureus* biofilm formation, no studies have directly tested the hypothesis that Nuc1 mediates the detachment and dispersal of cells from mature *S. aureus* biofilms.

In the present study, we constructed apparatus to measure *S. aureus* biofilm erosion and biofilm sloughing. Erosion refers to the continuous release of single cells or small clusters of cells from a biofilm at low levels over the course of biofilm formation, whereas sloughing refers to the sudden detachment of large portions of the biofilm, usually during the later stages of biofilm formation (18). We focused on Nuc1 because Nuc2 appears to play a minor role in MRSA biofilm formation (Fig. 3) and eDNA release (Fig. 4) under the conditions tested. Detachment by the *nuc1^−^* mutant was restored to WT levels by exogenous DNase (Fig. 5B) or a WT *nuc1* gene on a plasmid (Fig. 5C), confirming that Nuc1 mediates biofilm erosion under the conditions tested. In the biofilm sloughing assay, the *nuc1^−^* mutant released ten times as many CFUs as the WT strain (Fig. 6B), likely due to the increased biofilm biomass in these strains. The *nuc1^−^* mutant strain also released significantly larger cell aggregates than the WT strain (Fig. 6C and D; Table 2). The sizes of the detached cell aggregates in our study were similar to the sizes of aggregates that detached from *S. aureus* biofilms cultured in glass tubes perfused with the brain heart infusion medium (28) and in silicone tubing perfused with human plasma (29). Large, detached aggregates of *S. aureus* cells were previously shown to be highly resistant to antibiotics (28, 30) and phagocytosis (31, 32) and capable of initiating the colonization of endothelial cell layers under flow (29), which suggests that biofilm sloughing may contribute to metastatic infections associated with *S. aureus* (28).

Our results demonstrate that Nuc1 modulates biofilm erosion and sloughing in *S. aureus* USA300. A homologous nuclease was shown to modulate eDNA production, biofilm dispersal, and biofilm aggregate size in nontypeable *Haemophilus influenzae* (33). Since eDNA is a structural component of *Pseudomonas aeruginosa* biofilms (34), it is possible that Nuc1 may passively contribute to *P. aeruginosa* biofilm formation and dispersal during *S. aureus*/*P. aeruginosa* coinfection in the airways of cystic fibrosis patients (35) and in chronic wounds (36).
